# Predictive Role of Pretreatment Circulating miR-221 in Patients with Hepatocellular Carcinoma Undergoing Transarterial Chemoembolization

**DOI:** 10.3390/diagnostics13172794

**Published:** 2023-08-29

**Authors:** Nutcha Pinjaroen, Piyawan Chailapakul, Supachaya Sriphoosanaphan, Natthaya Chuaypen, Pisit Tangkijvanich

**Affiliations:** 1 Department of Radiology, Faculty of Medicine, Chulalongkorn University, Bangkok 10330, Thailand; nutcha.p@chula.ac.th; 2 Center of Excellence in Hepatitis and Liver Cancer, Department of Biochemistry, Faculty of Medicine, Chulalongkorn University, Bangkok 10330, Thailand; p.chailapakul@colostate.edu (P.C.); natthaya.ch56@gmail.com (N.C.); 3 Division of Gastroenterology, Department of Medicine, Faculty of Medicine, Chulalongkorn University, Bangkok 10330, Thailand; supachaya.sr@gmail.com

**Keywords:** hepatitis B virus, hepatocellular carcinoma, transarterial chemoembolization, microRNAs

## Abstract

Aberrantly expressed circulating microRNAs (miRNAs) have been demonstrated to have a crucial role in the diagnosis and prognostication of various cancers, including hepatocellular carcinoma (HCC). This research aimed to examine the role of specific miRNAs in predicting the outcomes for individuals with hepatitis B virus (HBV)-related HCC treated with transarterial chemoembolization (TACE). Stored serum specimens collected prior to the first TACE procedure were employed to determine the expression of serum miR-122, miR-221, and miR-224 using quantitative real-time PCR analysis. The study included 100 HCC patients (84% males, with an average age of 60 years) who were treated with TACE. Throughout the median follow-up spanning 18.5 months (within a range of 3 to 60 months), 42 (42.0%) patients met the criteria of TACE refractoriness. Through multivariate analysis, elevated expressed miR-221 (≥4.0 log10 copies) and advanced HCC staging were identified as independent factors related to TACE refractoriness and short overall survival. However, serum miR-122 and miR-224 levels were not linked to treatment response or overall survival. These findings underscored the potential of incorporating pretreatment levels of serum miR-221 into the established tumor staging to enhance the accurate assessment of TACE responsiveness and prognostic outcome of patients with HCC.

## 1. Introduction

Hepatocellular carcinoma (HCC) is one of the most aggressive cancers worldwide, with high prevalence in Southeast Asia, where chronic hepatitis B virus (HBV) infection is common [1]. In Thailand, the estimated incidence of HCC is 38.6 in men and 17.2 per 100,000 person-years in women [2]. Generally, the prognosis of HCC is considered poor due to an advanced stage at initial presentation, leading to unsuitability for curative therapy including surgical treatment and tumor ablation. In unresectable cases, transarterial chemoembolization (TACE) is the standard treatment for patients with intermediate HCC according to the Barcelona Clinic Liver Cancer (BCLC) guideline [3]. However, treatment response to TACE varies substantially due to the heterogeneous characteristics of HCC, as well as the extent of tumor burden, different etiological factors, hepatic functional reserve and the lack of reliable markers in predicting therapeutic outcome [4]. Repeated TACE is not recommended in patients who experience intrahepatic and extrahepatic progression associated with compromised liver function [5]. Thus, identifying patients who would benefit most from TACE is essential for improving the prognosis and optimizing survival outcome.

Although serum alpha-fetoprotein (AFP) has been regularly used for the diagnosis and monitoring of HCC, its performance in predicting prognosis is inadequate [6]. Thus, the identification of novel and effective predictive biomarkers in patients who would benefit most from TACE is necessary to improve treatment outcome of HCC. Recently, it has been demonstrated that the hepatoma arterial embolization prognostic (HAP) score is useful for predicting the treatment outcomes of TACE [7]. This model enables practitioners to classify patients into four groups based on albumin, bilirubin, AFP and tumor size. However, the HAP score still requires additional data among different patient populations and etiologic factors of HCC to verify its predictive value. MicroRNAs (miRNAs) are a class of small endogenous noncoding RNAs consisting of approximately 18–24 nucleotides that regulate gene expression by binding to specific mRNA targets and promoting their degradation and translational inhibition [8]. It has been revealed that miRNAs are associated with several biological processes, including development, differentiation, growth, homeostasis and immune activation [9]. Increasing evidence has indicated that aberrant miRNA expression plays an essential role in the pathogenesis of several cancers, including HCC [10,11]. Moreover, circulating miRNAs have been shown as potential biomarkers for HCC due to their tissue-specific expression and stability in body fluids [10,12].

Among them, miR-122 is a hepatic-specific miRNA that displays a key role involving several functions of the liver [13] and has been shown to be essential in hepatic carcinogenesis by acting as a tumor suppressor [14]. In this context, previous data reported that the loss of miR-122 in knockout mice was subsequently associated with the development of liver cancer [14]. In addition, circulating miR-122 was previously shown as a potential biomarker for detecting HBV-related HCC [15]. Among other HCC-related miRNAs, miR-221 is one of the consistently upregulated miRNAs in cancerous tissues [16]. It was shown that miR-221 had an oncogenic function in promoting HCC proliferation by controlling cell-cycle inhibitors [17,18]. In addition, the upregulated expression of circulating miR-221 correlated with tumor stage and was independently associated with poor prognosis of HCC [19]. Besides their diagnostic and prognostic utility, emerging evidence also demonstrates the potential role of targeting miR-122 and/or miR-221 as an innovative therapeutic strategy for HCC in an animal model [20]. Regarding miR-224, deregulation of this miRNA was observed in the pre-malignant lesions and accumulated throughout the multistep process of HBV-related HCC [21]. A recent report also showed that circulating miR-224 might be a sensitive biomarker for screening and monitoring HCC [22]. In addition, high tissue expression of miR-224 showed a significant correlation with poor overall survival rates [23].

Currently, limited information is available regarding the predictive role of circulating miR-122, miR-221 and miR-224 in patients with HCC treated with TACE. Thus, this study was aimed at investigating the association of their baseline expression levels in predicting TACE refractoriness and overall survival of Thai patients with HBV-related HCC.

## 2. Materials and Methods

### 2.1. Patients and Samples

Serum samples for miR-122, miR-221 and miR-224 quantification were obtained from patients who were diagnosed with HBV-related HCC and underwent TACE for the first time between August 2010 and September 2016 at King Chulalongkorn Memorial Hospital, Bangkok, Thailand. The diagnosis of HCC was based on typical imaging modalities and/or histopathology according to the international guideline [3]. Briefly, the criteria of HCC included focal hepatic lesions with hyperattenuation at the arterial phase and hypoattenuation at the portal phase in dynamic computed tomography (CT) or magnetic resonance imaging (MRI). Liver biopsy or fine needle aspiration was conducted to confirm the diagnosis in cases with atypical imaging features. The baseline clinical characteristics of patients with HCC were collected. HCC staging was classified according to the BCLC staging system [3]. Additionally, the overall survival of each patient, described by the time interval between initial enrollment and death or the last follow-up visit, was documented. All patients had serum hepatitis B surface antigen (HBsAg) positivity. Patients with hepatitis C virus (HCV) and/or HIV co-infection were excluded. Patients were also excluded if they had been previously treated using other therapeutic modalities for HCC.

### 2.2. TACE Procedure

TACE was performed by interventional radiologists. After hepatic angiography was achieved, a microcatheter was selectively inserted into the feeding artery. Chemoembolization then was performed. An emulsion of Lipiodol (Geurbet, Villepinte, France) mixed with Mitomycin C was infused, followed by chemoinfusion with Fluorouracil (5-FU). The given doses depended on the tumor size, the position of the catheter, arterial blood flow and the patient’s liver function. The usual dose of Lipiodol was 10 mL, ranging from 2.5 to 20 mL. The maximum doses of chemotherapeutic agents were 20 mg of Mitomycin C and 500 mg of 5-FU. Embolic materials such as gelfoam, polyvinyl alcohol (PVA) or spherical particles may be required to achieve flow stasis of the feeding artery. After TACE, angiography was performed to determine tumor devascularization, extent of arterial occlusion and residual disease.

All patients underwent follow-up evaluations 4–8 weeks after TACE with multiphasic-CT or MRI, together with routine monitoring of the complete blood count (CBC), coagulation profile, liver function tests, renal function and serum AFP levels. The evaluation of tumor response was defined according to the modified Response Evaluation Criteria in Solid Tumors (mRECIST). In responders, repeat TACE was performed with a time interval of at least 4 weeks. TACE refractoriness was defined using the following criteria: (1) two or more consecutive insufficient responses to TACE (defined as viable tumor >50%) even after changing chemotherapeutic agents and/or re-analysis of the feeding artery; (2) the appearance of a higher number of new intrahepatic lesions than previously recorded; (3) the appearance of vascular invasion or extrahepatic spread; (4) the deterioration of liver function as indicated by an increase in the Child–Pugh score ≥ 2 points; and (5) progressively elevated levels of conventional tumor markers [24].

This study was conducted according to the Declaration of Helsinki for the participation of human individuals. The protocol was approved by the Ethics Committee, Faculty of Medicine, Chulalongkorn University, and written informed consent was obtained from each participant. Serum samples collected prior to the 1st TACE were separated by centrifugation and stored at −70 °C until further analysis.

### 2.3. Isolation and Reverse Transcription of Circulating miRNAs

Stored sera were assessed for miR-122, miR-221 and miR-224 expressions. First, total miRNA was extracted from serum samples with miRNA Isolation Kit (Geneaid Biotech Ltd., New Taipei City, Taiwan) according to the manufacturer’s instructions. Approximately 5 μg of miRNA was then reverse-transcribed into cDNA by means of the TaqMan^®^ microRNA RT kit (Applied Biosystems, Foster City, CA, USA) and miRNA-specific stem-loop primers (Applied Biosystems, Foster City, CA, USA) using thermal cycler PCR conditions (pre-denaturation at 16 °C for 30 min, primer extension with the first cDNA strand synthesis at 42 °C for 30 min, and reaction termination at 85 °C for an additional 5 min).

### 2.4. Real-Time Quantitative PCR Assay

Real-Time qPCR of cDNA was amplified and evaluated by means of the TaqMan^®^ miRNA assay (Applied Biosystems, Foster City, CA, USA) has-miR-122 (Assay ID. 002245), has-miR-221 (Assay ID. 000524) and has-miR-224 (Assay ID. 002099) using a StepOnePlus^TM^ Real-Time PCR machine (Applied Biosystems, Foster City, CA, USA) with the following conditions: holding step at 50 °C for 2 min and 95 °C for 10 min, followed by 40 cycles of denaturation at 95 °C for 15 s, annealing and extension at 60 °C for 1 min. miRNA target sequences are provided in Supplementary Table S1. The cycle threshold (Ct) values of miRNAs were converted into the number of microRNA copies using the standard curve from a serial dilution (10^2^–10^10^ copies/µL) of plasmid miR-122, miR-221 and miR-224.

### 2.5. Statistical Analysis

Data were presented as the mean ± standard deviation (SD), median (range) and percentages as appropriate. Statistical comparisons between continuous variables were undertaken using independent-sample t-tests, while categorical variables were assessed via the χ2 test. The correlation between miRNA expressions and baseline clinical variables was evaluated using the χ2 test or Spearman’s rank test as appropriate. The median score of miRNAs were used to stratify patients into high- and low-expression groups, and Kaplan–Meier analysis with a log-rank test was used to evaluate the overall survival difference between two difference expression groups. Univariate and multivariate logistic regression analyses were used to predict categorical variables. A *p*-value < 0.05 was statistically significant. All statistical analyses were performed using the SPSS 22.0 software (SPSS Inc., Chicago, IL, USA).

## 3. Results

### 3.1. Baseline Characteristics

Table 1 shows the baseline clinical characteristics of the 100 patients in this study. The median age was 60.5 years (range 30–83 years), and 84% of patients were male. Most patients had underlying compensated cirrhosis (Child-Pugh A, 75%) with BCLC stages of A-B (58%). The median levels of serum miR-122, miR-221 and miR-224 at baseline were 4.1, 4.0 and 1.5 log_10_ copies, respectively. The median follow-up period after the first TACE was 18.5 months (range 3–60 months).

### 3.2. Correlation of miRNA Expression with Baseline Parameters

**Figure 1:**
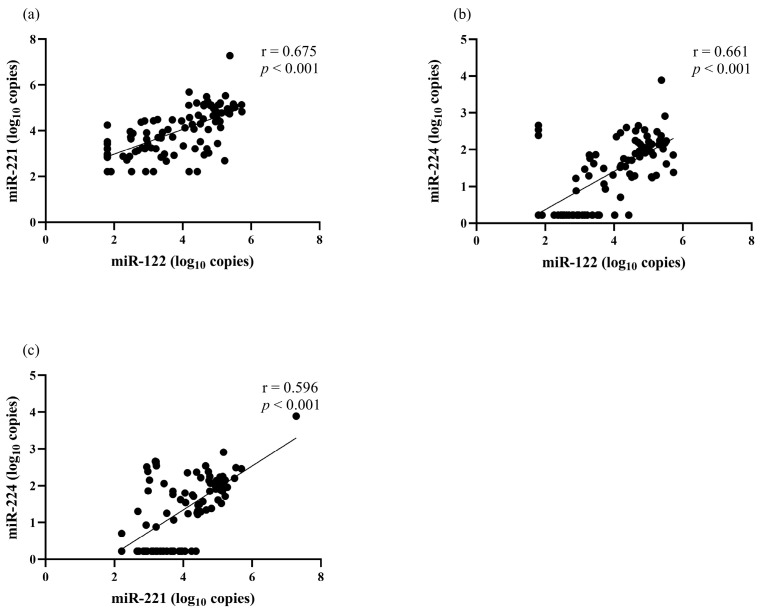
The correlation among serum miRNAs levels at baseline. (**a**) miR-122 and miR-221, (**b**) miR-122 and miR-224, (**c**) miR-221 and miR-224.

The correlation between serum miRNA levels and clinical parameters was analyzed. There was a positive correlation between miR-122 and miR-221 (r = 0.675, *p* < 0.001), miR-122 and miR-224 (r = 0.661, *p* < 0.001), as well as miR-221 and miR-224 (r = 0.596, *p* < 0.001) (Figure 1). Serum miR-122 correlated with the BCLC stage but did not correlate with other clinical parameters, including sex, age, biochemical parameters, AFP level, Child–Pugh classification and tumor size (Table 2). Serum miR-221 level had a positive correlation with total bilirubin level (r = 0.310, *p* = 0.002) but did not have a significant correlation with other parameters. For serum miR-224, its level did not correlate with any baseline clinical parameters.

### 3.3. Baseline Predictive Factors for Overall TACE Refractoriness

During the follow-up period, 58 (58.0%) patients achieved a response and 42 (42.0%) patients experienced overall TACE refractoriness following repetitive TACE treatments. Serum miR-122 miR-221 and miR-224 expression were entered into univariate and multivariate regression analyses with other baseline parameters that could influence treatment outcome. In univariate analysis, our data demonstrated that high AFP (≥400 ng/mL), large tumor size (≥ 5 cm), BCLC stage C, low serum miR-122 level (<4.1 log_10_ copies) and high serum miR-221 level (≥4.0 log_10_ copies) were predictive factors for overall TACE refractoriness in patients with HCC. In multivariate analysis, the BCLC stage and serum miR-221 level were detected as being independent factors for overall TACE refractoriness (Table 3).

### 3.4. Baseline Predictive Factors of Overall Survival

**Figure 2:**
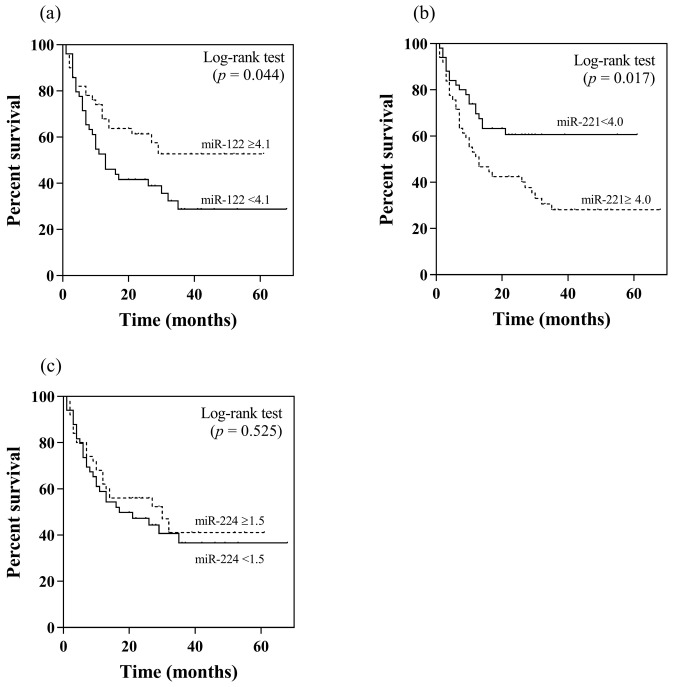
Kaplan–Meier analysis of overall survival in relation to pretreatment serum miRNA levels by using their median values as the cut-off points: (**a**) miR-122, (**b**) miR-221, (**c**) miR-224.

In this study, patients with and without TACE refractoriness had an overall survival of 15.5 and 42.9 months, respectively (*p* < 0.001). Apart from their association with TACE refractoriness, the potential prognostic roles in terms of overall survival of serum miR-122, miR-221 and miR-224 were further analyzed. Using their median values as the cut-off levels, patients with a high serum miR-122 level (≥4.1 log_10_ copies) had higher overall survival compared to individuals with a low level (<4.1 log_10_ copies) (37.5 vs. 27.9 months, *p* = 0.044 by log rank test) (Figure 2a). Regarding serum miR-221, patients with a low level (<4.0 log_10_ copies) exhibited a better overall survival than those with a high serum level (≥4.0 log_10_ copies) (40.2 vs. 27.4 months, *p* = 0.017) (Figure 2b). For serum miR-224, there was no difference in overall survival between patients who had high or low levels (32.6 vs. 32.3 months, *p* = 0.525) (Figure 2c).

Additionally, univariate analysis was performed to investigate predictive factors for overall survival. These baseline factors included age, gender, AFP level, Child–Pugh classification, HAP score, TACE refractoriness, BCLC stage and serum miRNA levels. Among these parameters, AFP, Child–Pugh, tumor size, BCLC stage and serum miR-221 level were selected for multivariate analysis. Based on multivariate analysis, our data demonstrated that BCLC stage and serum miR-221 level were independently associated with overall survival (Table 4).

## 4. Discussion

An increasing number of recent studies have indicated that the dysregulation of miRNAs is closely linked to malignant transformation and HCC progression in patients with underlying chronic liver disease [11]. Considering their accessibility and high stability in the circulation, detecting miRNAs in plasma/serum is applicable for the diagnosis and prognosis of HCC. Indeed, accurate predictors in selecting patients for initiation and retreatment with TACE is essential for obtaining an optimal survival outcome [4]. In this study, we aimed at evaluating the prognostic role of circulating miRNAs in patients with HBV-related HCC undergoing TACE. To achieve this objective, frequently reported miRNAs including miR-122, miR-221 and miR-224 were selected. miR-122 is considered to be a liver-specific miRNA, while miR-221 and miR-224 have been shown to be consistently upregulated in serum and liver tissue specimens of patients with HBV-related HCC [16]. In this report, our data showed that low levels of circulating miR-122 and high miR-221 were predictive of TACE failure/refractoriness in univariate analysis. However, only high circulating miR-221 expression was independently associated with TACE refractoriness and poor overall survival in patients who were undergoing TACE.

miR-221, encoded by chromosome Xp11.3, is over-expressed in many types of cancer by acting as an oncogene or tumor-suppressive gene. It has been shown that miR-221 promotes the tumor progression of several cancers, including breast, prostate, and bladder cancers [25]. On the contrary, this miRNA could inhibit tumor development in pancreatic and ovarian cancers. Collectively, it is speculated that the role of miR-221 might not be similar in various cancer types [25]. Regarding HCC, miR-221 is among the most consistently up-regulated of all miRNAs that have been studied in this type of liver cancer. Several reports demonstrated that miR-221 was overexpressed in HCC tissue as compared with their adjacent non-cancerous liver tissue [26,27,28]. A previous in vivo report revealed that miR-221 overexpression promoted tumor growth and invasion through the modulation of the mTOR pathway via DNA damage-inducible transcript 4 (DDIT4) [27]. Moreover, overexpression of miR-221 was shown to induce HCC proliferation via the cyclin-dependent kinase inhibitors CDKN1B/p27 and CDKN1C/p57, as well as modulating Bmf, a proapoptotic BH3-only protein [29]. Additionally, targeting miR-221 was capable of reducing HCC cell proliferation, increasing cell-cycle arrest and improving survival in an animal model [18]. Together, these data indicate that miR-221 is a key oncogenic miRNA that plays an essential role in hepatocarcinogenesis and tumor progression through multiple signaling pathways related to proliferation and apoptosis.

Previous data showed that monitoring the serum miR-221 level could be of clinical significance as a potential diagnostic tool and for improving the treatment efficacy of HCC. For instance, a recent report also identified circulating miR-221 in a panel of three miRNAs as a reliable noninvasive biomarker for the diagnosis of HCC [30]. It was also shown that this miRNA was elevated by approximately 5-fold in patients with HCC compared with those without HCC. Moreover, circulating miR-221 expression was positively correlated with cirrhosis, tumor size and tumor stage [19]. In the present study, a correlation between serum miR-221 and the severity of underlying liver disease, as well as tumor stage, was not observed. The reason for this discrepancy between studies is unclear, but might be related to diverse clinical background, as in the above-mentioned study, included approximately one-third of participants had non-HBV-related HCC. Whether serum miR-221 is significantly upregulated in HCC regardless of the underlying etiological causes of HCC needs to be further investigated.

Notably, our data demonstrate for the first time that high baseline miRNA-221 expression is associated with TACE refractoriness. Considering the clinical benefits of several novel molecular target agents, conversion from TACE to systemic therapies at the time of TACE refractoriness/failure may be an appropriate decision, with a greater impact on survival time [5]. Our results regarding multivariate regression analysis also indicated that a high serum miR-221 level was associated with poor overall survival in patients with HCC undergoing TACE. However, HAP score did not exhibit a predictive value for overall survival in multivariate analysis. Thus, the incorporation of novel biomarkers at baseline such as circulating miRNAs might provide a better prediction of treatment response and prognosis following TACE. In line with our findings, previous studies revealed that HCC patients undergoing surgical resection with high serum miR-221 levels had a shorter survival time than those with low expression [19,26]. Collectively, these data might suggest that circulating miR-221 could be an independent predictor of survival and can therefore serve as a potential biomarker for predicting the outcome of various treatment modalities in patients with HCC.

Accumulating evidence has suggested that the dysregulation of miR-122 is linked to malignant transformation and HCC development [13]. In fact, the expression of miR-122 is typically down-regulated in HBV-related HCC tissue, suggesting that this miRNA could act as a tumor suppressor by regulating HCC growth, invasion and angiogenesis, as well as by enhancing apoptosis and cell cycle arrest [16]. In this study, our data revealed that baseline circulating miR-122 levels were predictive of overall TACE refractoriness in univariate analysis. In multivariate analysis, however, serum miR-122 was not selected as an independent predictor of overall TACE refractoriness. These results were not in line with a previous report conducted in South Korea, which demonstrated that a high pretreatment miR-122 level predicted TACE refractoriness [31]. This discrepancy between studies could be explained by several factors including differences in patient populations, the inclusion criteria, the definitions of TACE refractoriness, discrepancies in methodological protocols and the heterogeneity of HCC [32]. Moreover, inconsistent expression of circulating miR-122 was frequently reported among various studies in patients with HBV-related HCC, as it was shown to be up-regulated in some reports but was down-regulated in others [15,33,34]. In addition, circulating miR-122 level was not independently predictive of overall survival in our study, which was also contradictory to the above-mentioned report.

Regarding miR-224, this miRNA was shown to be up-regulated in cancerous liver tissues and involved in tumorigenesis in in vitro experiments [23,35]. Additionally, high expression of miR-224 in liver tissue specimens displayed significant correlation with HBV infection and poor overall survival in patients with HCC [23]. Moreover, circulating miR-224 was selected as a potential sensitive biomarker in discriminating HCC from those without liver cancer [22]. However, the prognostic role of circulating miR-224 in patients with HCC undergoing tumor therapy is less well defined. In this report, our data did not support the clinical benefit of miR-224 in terms of predicting the therapeutic outcome and overall survival of patients with HCC undergoing TACE.

Our study might have some limitations. First, this retrospective study was conducted in a tertiary hospital, and thus a selection bias involving patient enrollment might be possible. Second, we enrolled only patients with HBV-related HCC, and it is uncertain whether our findings could be extended to other causes of HCC such as HCV and fatty liver diseases. Finally, we did not assess other miRNAs such as miR-200a, miR-335, miR-210, miR-199a/b-3p and miR-373 that could predict the efficacy of TACE in patients with HCC in other reports [36,37,38,39,40]. To address this limitation, additional studies are necessary to explore the predictive role of circulating miRNA expression in other populations with different etiological factors of HCC.

## 5. Conclusions

In summary, our data indicated that pretreatment serum miR-221 level could serve as a potential biomarker to optimize candidate selection for TACE. Thus, the incorporation of this miRNA into the conventional staging systems might provide a better prediction of treatment outcome and prognosis of patients with HCC undergoing TACE.

## Figures and Tables

**Table 1:** Baseline characteristics of the patients in this study.

Parameters	*n* = 100
Gender (Male/Female)	84 (84)/16 (16)
Age (years)	60.5 (30–83)
Aspartate aminotransferase, IU/L	59.5 (20–247)
Alanine aminotransferase, IU/L	43.0 (15–207)
Albumin, g/L	3.7 (2.2–4.7)
Total bilirubin, mg/dL	1.0 (0.2–2.6)
Alpha fetoprotein, ng/mL	80 (0.9–500,000)
Cirrhosis	93 (93)
Child-Pugh Classification (A/B)	75 (75)/18 (18)
Tumor size, cm	6.5 (1.4–14.0)
BCLC stage (A/B/C)	32 (32)/26 (26)/42 (42)
HAP score (A/B/C/D)	49 (49)/22 (22)/24 (24)/5 (5)
miR-122, log_10_ copies	4.1 (1.8–5.7)
miR-221, log_10_ copies	4.0 (2.2–5.7)
miR-224, log_10_ copies	1.5 (0.2–3.9)
Follow-up duration after 1st TACE, months	18.5 (3–60)

Data expressed as median (range) or *n* (%) as appropriate.

**Table 2:** Correlation of miRNA expression and baseline characteristics.

	miR-122 Levels			miR-224 Levels			miR-221 Levels		
	<4.1 log_10_ Copies	≥4.1 log_10_ Copies	*p*	<4.0 log_10_ Copies	≥4.0 log_10_ Copies	*p*	<1.5 log_10_ Copies	≥1.5 log_10_ Copies	*p*
Age (year)			0.841			0.841			0.688
<60	23 (51.1)	21 (48.9)		22 (53.3)	23 (51.1)		24 (53.3)	21 (46.7)	
≥60	27 (49.1)	24 (50.9)		28 (50.9)	27 (49.1)		26 (47.3)	29 (52.7)	
Gender			0.414			0.171			0.786
Male	44 (52.4)	40 (47.6)		39 (46.4)	45 (53.6)		43 (51.2)	41 (48.8)	
Female	6 (37.5)	10 (62.5)		11 (68.8)	5 (31.2)		7 (43.8)	9 (56.3)	
AFP (ng/mL)			0.408			0.408			0.214
<400	29 (46.0)	34 (54.0)		34 (54.0)	29 (46.0)		28 (44.4)	35 (55.6)	
≥400	21 (56.8)	16 (43.2)		16 (43.2)	21 (56.8)		22 (59.5)	15 (40.5)	
Child-Pugh			0.436			0.795			0.192
0–A	43 (52.4)	39 (47.6)		40 (48.8)	42 (51.2)		44 (53.7)	38 (46.3)	
B	7 (38.9)	11 (61.1)		10 (55.6)	8 (44.4)		6 (33.3)	12 (66.7)	
Tumor (cm)			0.537			0.303			1.000
<5 cm	17 (44.7)	21 (55.3)		22 (57.9)	16 (42.1)		19 (50.0)	19 (50.0)	
≥5 cm	33 (53.2)	29 (46.8)		28 (45.2)	34 (54.8)		31 (50.0)	31 (50.0)	
BCLC stage			0.043 *			0.840			0.544
A–B	24 (41.4)	34 (58.6)		30 (51.7)	28 (48.3)		27 (46.6)	31 (53.4)	
C	26 (51.9)	16 (38.1)		20 (47.6)	22 (52.4)		23 (54.8)	19 45.2)	

Data expressed as *n* (%), * *p*-value < 0.05.

**Table 3:** Univariate and multivariate analyses for predicting TACE refractoriness.

Baseline Parameters	Univariate Analysis		Multivariate Analysis	
	OR (95% CI)	*p* Value	OR (95% CI)	*p* Value
Age (≥60 vs. <60 years)	1.98 (0.88–4.44)	0.097		
Gender (male vs. female)	2.48 (0.74–8.31)	0.142		
AFP (≥400 vs. <400 ng/mL)	3.81 (1.62–8.95)	0.002 *	1.32 (0.43–4.07)	0.626
Child-Pugh (B vs. 0–A)	1.95 (0.70–5.47)	0.203		
BCLC stage (C vs. A/B)	9.58 (3.81–24.12)	<0.001 *	7.69 (2.41–24.55)	0.001 *
HAP score (C–D vs. A–B)	5.36 (2.23–12.85)	<0.001 *	2.63 (0.84–8.25)	0.097
miR-122, log_10_ copies (<4.1 vs. ≥4.1)	2.74 (1.20–6.23)	0.016 *	0.96 (0.27–3.36)	0.945
miR-221, log_10_ copies (≥4.0 vs. <4.0)	3.27 (1.42–7.52)	0.005 *	4.95 (1.32–18.46)	0.017 *
miR-224, log_10_ copies (<1.5 vs. >1.5)	1.64 (0.74–3.66)	0.225		

OR, odds ratio; CI, confidence interval; * *p*-value < 0.05.

**Table 4:** Univariate and multivariate analyses for predicting poor overall survival.

Baseline Parameters	Univariate Analysis		Multivariate Analysis	
	OR (95% CI)	*p* Value	OR (95% CI)	*p* Value
Age (≥60 vs. <60 years)	1.32 (0.77–2.26)	0.318		
Gender (male vs. female)	1.53 (0.78–2.97)	0.214		
AFP (≥400 vs. <400 ng/mL)	3.35 (1.93–5.80)	<0.001 *	1.56 (0.84–2.87)	0.158
Child-Pugh (B vs. 0–A)	1.95 (1.02–3.75)	0.045 *	1.34 (0.66–2.68)	0.418
BCLC stage (C vs. A/B)	6.11 (3.37–11.07)	<0.001 *	3.59 (1.74–7.42)	0.001 *
HAP score (C–D vs. A–B)	4.80 (2.60–8.86)	<0.001 *	2.76 (1.35–5.63)	0.005 *
TACE refractoriness (yes vs. no)	5.22 (2.92–9.31)	<0.001 *	2.42 (1.21–4.88)	0.013 *
miR-122, log_10_ copies (<4.1 vs. ≥4.1)	1.74 (1.00–3.02)	0.050	0.77 (0.38–1.55)	0.001 *
miR-221, log_10_ copies (≥4.0 vs. <4.0)	1.99 (1.10–3.43)	0.022 *	2.10 (1.03–4.28)	0.466
miR-224, log_10_ copies (<1.5 vs. >1.5)	1.19 (0.69–2.04)	0.532		

OR, odds ratio; CI, confidence interval; * *p*-value < 0.05.

## Data Availability

The data used in the study are available from the corresponding author on reasonable request.

## Supplementary Materials

The following supporting information can be downloaded at: https://www.mdpi.com/article/10.3390/diagnostics13172794/s1, Table S1: List of TaqMan^®^ miRNAs and target sequence used in this study.
