# Supplementary material for: Predictive Role of Pretreatment Circulating miR-221 in Patients with Hepatocellular Carcinoma Undergoing Transarterial Chemoembolization
Source: Diagnostics (Basel). 2023 Aug 29;13(17):2794. doi: 10.3390/diagnostics13172794 (PMC10487177; doi:10.3390/diagnostics13172794)
Supplement: Supplementary file 1 [file diagnostics-13-02794-s001.zip › diagnostics-2515194-supplementary.pdf]

Table S1. List of TaqMan® miRNAs and target sequence used in this study.

miRNA	Assay ID	Target sequence
hsa-miR-122	002245	UGGAGUGUGACAAUGGUGUUUG
hsa-miR-221	000524	AGCUACAUUGUCUGCUGGGUUUC
hsa-miR-224	002099	CAAGUCACUAGUGGUUCCGUU
